# Beyond the Horizon: A Comprehensive Review of Contemporary Strategies in Sepsis Management Encompassing Predictors, Diagnostic Tools, and Therapeutic Advances

**DOI:** 10.7759/cureus.64249

**Published:** 2024-07-10

**Authors:** Pavithra Konjety, Vivek G Chakole

**Affiliations:** 1 Anaesthesiology, Jawaharlal Nehru Medical College, Datta Meghe Institute of Higher Education and Research, Wardha, IND; 2 Research, Jawaharlal Nehru Medical College, Datta Meghe Institute of Higher Education and Research, Wardha, IND

**Keywords:** telemedicine, global health disparities, antibiotic resistance, artificial intelligence, precision medicine, sepsis management

## Abstract

This comprehensive review offers a detailed exposition of contemporary strategies in sepsis management, encompassing predictors, diagnostic tools, and therapeutic advances. The analysis elucidates the dynamic nature of sepsis, emphasizing the crucial role of early detection and intervention. The multifaceted strategies advocate for a holistic and personalized approach to sepsis care from traditional clinical methodologies to cutting-edge technologies. The implications for clinical practice underscore clinicians' need to adapt to evolving definitions, integrate advanced diagnostic tools, and embrace precision medicine. Integrating artificial intelligence and telemedicine necessitates a commitment to training and optimization. Judicious antibiotic use and recognition of global health disparities emphasize the importance of a collaborative, global effort in sepsis care. Looking ahead, recommendations for future research underscore priorities such as longitudinal studies on biomarkers, precision medicine trials, implementation science in technology, global health interventions, and innovative antibiotic stewardship strategies. These research priorities aim to contribute to transformative advancements in sepsis management, ultimately enhancing patient outcomes and reducing the global impact of this critical syndrome.

## Introduction and background

Sepsis is a complex and dynamic syndrome characterized by a dysregulated host response to infection, leading to organ dysfunction. It represents a continuum of severity, ranging from sepsis to severe sepsis and septic shock [1]. The clinical manifestations of sepsis include a systemic inflammatory response, organ dysfunction, and, in severe cases, a life-threatening drop in blood pressure [2]. Understanding sepsis requires recognizing its underlying pathophysiology, where the immune response, microbial factors, and host genetics intertwine. The definition has evolved, reflecting advances in scientific understanding. The recent emphasis on organ dysfunction as a key component has refined our approach to identifying and managing sepsis [3].

Sepsis stands as a leading cause of morbidity and mortality worldwide, exerting a substantial burden on healthcare systems. The timely and effective management of sepsis is pivotal in preventing its progression to severe forms, reducing mortality rates, and minimizing long-term sequelae for survivors. Failure to recognize and intervene promptly can lead to a cascade of events culminating in multiple organ failures, underscoring the critical importance of a proactive and well-informed approach to sepsis management [4]. The impact of sepsis extends beyond individual health outcomes, affecting communities, healthcare infrastructures, and global public health. Addressing sepsis requires a multifaceted understanding of its clinical, epidemiological, and societal implications [5].

This review aims to synthesize current knowledge on sepsis management, emphasizing predictors, diagnostic tools, and therapeutic advances. By examining the latest research findings and technological innovations, the review seeks to provide a comprehensive overview for clinicians, researchers, and healthcare policymakers. The scope encompasses a broad array of topics, from traditional clinical approaches to emerging technologies, offering a holistic perspective on the evolving landscape of sepsis management.

## Review

Predictors of sepsis

Identification of Risk Factors

Age, comorbidities, and immunocompromised states: Research has focused on predicting sepsis and identifying associated risk factors, particularly through machine learning models and artificial intelligence (AI). Studies have predominantly concentrated on the early prediction and detection of sepsis, utilizing datasets like the Medical Information Mart for Intensive Care (MIMIC) and the University of California San Francisco Medical Center database [6-8]. AI algorithms have been developed to forecast sepsis risk preemptively, leveraging unstructured healthcare data [9]. Moreover, biomarkers and personalized medicine have emerged as pivotal strategies in sepsis therapy, facilitating targeted treatments for individuals likely to benefit [9]. Despite advancements, predicting and identifying sepsis early remains challenging, with current tools leaving significant gaps in our ability to fully ascertain at-risk patients [7]. Further research and development are necessary to enhance the accuracy and timeliness of sepsis prediction and risk factor identification.

Nosocomial infections: Nosocomial infections pose a substantial risk for sepsis, particularly in intensive care units (ICUs). Studies have pinpointed several predictive risk factors for nosocomial infections, including surveillance disparities, inadequate infrastructure, and insufficient personnel training [10,11]. Machine learning models and AI promise to predict sepsis risk, leveraging unstructured healthcare data [8,9]. Nonetheless, accurately identifying and predicting sepsis early remains challenging, with current tools leaving significant gaps in our ability to comprehensively identify at-risk patients [7]. Achieving precise and early sepsis recognition linked to timely interventions remains a crucial yet elusive objective for developers of bedside electronic decision support systems [7].

Genetic predispositions: Genetic factors play a critical role in sepsis development. Several studies have identified genetic variations associated with increased sepsis risk, such as polymorphisms in genes like tumor necrosis factor (TNF), suggesting potential applications for identifying high-risk patients [12]. Additionally, sepsis is recognized as a polygenic disease, with multiple genetic variants across immune and coagulation proteins influencing susceptibility [13,14]. Polygenic risk scores have shown promise in predicting sepsis risk, with specific genetic variants like NOS2, PPARG, HSPA12A, and TLR1 associated with increased susceptibility [15,16]. These findings underscore the importance of understanding genetic predispositions to sepsis and their implications for risk assessment and personalized medicine.

Biomarkers as Predictive Tools

Procalcitonin (PCT): PCT is a promising biomarker for predicting sepsis. In a prospective observational analysis, PCT, alongside other biomarkers, demonstrated outstanding performance in predicting 28-day all-cause mortality among patients diagnosed with sepsis or septic shock, surpassing the Sequential Organ Failure Assessment (SOFA) score [17]. Additionally, PCT, along with biomarkers like N-terminal pro B-type natriuretic peptide (NT-proBNP), interleukin-6 (IL-6), prothrombin time (PT), and thrombin time (TT), is supportive in early sepsis diagnosis and in assessing its progression and prognosis [18]. Another study highlighted PCT's prognostic significance in predicting 28-day mortality in critically ill sepsis patients, underscoring its potential value in early mortality prediction [19]. These findings underscore PCT's utility as a valuable biomarker in predicting and early identifying sepsis, offering potential benefits for patient management and outcomes.

C-reactive protein (CRP): CRP has been studied as a biomarker for sepsis, but its predictive utility is limited. Recent research has suggested that the CRP-to-albumin ratio (CAR) can be a new predictor for conditions like pneumonia, stroke, and certain cancers [20]. However, CRP lacks specificity for sepsis and can be elevated in other inflammatory conditions, thus limiting its diagnostic usefulness for sepsis [21]. In contrast, PCT has shown promise as a predictive biomarker for sepsis, demonstrating excellent performance in predicting 28-day all-cause mortality among patients with sepsis or septic shock [17,22]. Additionally, PCT supports early sepsis diagnosis and helps evaluate its progression and prognosis [21]. Biomarker kinetics, rather than single values, are crucial for predicting sepsis and assessing antibiotic therapy response [21]. While CRP may offer some predictive value for sepsis, PCT is a more promising biomarker for sepsis prediction and early identification.

Other emerging biomarkers: Several emerging biomarkers show potential for predicting sepsis. For instance, low absolute lymphocyte counts predict postoperative sepsis and bacteremia better than conventional markers like CRP [21]. Additionally, a wide array of sepsis biomarkers have been identified, including fluid-phase pattern recognition molecules (PRMs), cytokines, chemokines, and damage-associated molecules [23]. Biomarker kinetics are crucial for predicting sepsis and assessing antibiotic therapy response, aiding diagnosis and treatment evaluation [22]. Furthermore, a prospective observational analysis highlighted the exceptional predictive performance of pentraxin 3 (PTX-3), IL-6, PCT, and lactate in predicting 28-day all-cause mortality in sepsis or septic shock patients, outperforming the SOFA score [22]. These findings underscore the potential of various emerging biomarkers in predicting and managing sepsis, providing valuable insights into early diagnosis, prognosis, and treatment.

Diagnosis of sepsis

Clinical Assessment

Systemic inflammatory response syndrome (SIRS) criteria: The SIRS criteria have historically been a screening tool for identifying patients with potential sepsis. These criteria encompass four parameters: temperature >38°C or <36°C, heart rate >90 beats/min, respiratory rate >20 breaths/min, and abnormal white blood cell count (either >12,000/mm³ or <4,000/mm³ or >10% bands) [2,24,25]. However, the Sepsis-3 guidelines have moved away from using SIRS due to its high sensitivity and lack of specificity [25,24]. Instead, the guidelines recommend employing the quick SOFA (q-SOFA) score, which includes altered mental status, respiratory rate >22 breaths/min, and systolic blood pressure <100 mm Hg as criteria for identifying patients at risk for sepsis [26]. Emerging biomarkers like PCT show promise in predicting sepsis and may assist in early diagnosis and evaluating disease progression and prognosis [2,26,27]. Biomarker kinetics, rather than single values, are emphasized for their utility in predicting sepsis and assessing response to antibiotic therapy [25].

SOFA score: The SOFA score is a comprehensive tool used to assess the severity of organ dysfunction in sepsis patients. This score ranges from 0 to 24 points, with higher scores indicating more severe organ dysfunction [28,29]. It evaluates multiple organ systems, including respiratory, coagulation, liver, cardiovascular, central nervous, and renal [28]. The q-SOFA score, a simplified version of SOFA, assesses three parameters: respiratory rate, altered mental status, and systolic blood pressure [28]. Scores range from 0 to 3, with higher scores indicating more severe organ dysfunction [28]. Both SOFA and q-SOFA scores are utilized in diagnosing sepsis and predicting patient prognosis [28]. As measured by the area under the curve (AUC), their diagnostic capabilities are reported as 0.805 for SOFA, 0.763 for q-SOFA, and 0.856 for their combination in diagnosing sepsis [28]. When assessing patient condition, the AUC values for SOFA, q-SOFA, and the change in SOFA score (ΔSOFA) are 0.759, 0.716, and 0.685, respectively, with a combined AUC of 0.786 [28]. For predicting prognosis, the AUC values are 0.782 for SOFA, 0.753 for q-SOFA, 0.714 for ΔSOFA, and 0.929 for their combined use [28]. Both scores are valuable in clinical practice for their diagnostic and prognostic utility, with q-SOFA offering a simplified yet similarly effective alternative to SOFA.

Advanced Diagnostic Techniques

Molecular diagnostics: Molecular diagnostic techniques represent a promising approach in sepsis diagnosis by enabling rapid pathogen identification and detection of organisms often missed by conventional methods. Techniques like polymerase chain reaction (PCR) and real-time PCR offer broad-spectrum amplification capabilities for pathogen detection [30-32]. Biomarkers such as PCT and the CD64 index have also demonstrated good diagnostic accuracy in identifying sepsis [23,33]. Despite these advancements, blood culture analysis remains the gold standard for sepsis diagnosis due to its comprehensive nature [32]. The ideal sepsis diagnostic test should combine broad-based detection, high sensitivity and specificity, polymicrobial pathogen identification, and detection of drug resistance [32]. These findings underscore the potential of molecular diagnostics and biomarkers in enhancing early sepsis detection and treatment.

Imaging modalities: Imaging modalities play a limited role in sepsis diagnosis but are valuable for identifying infection sources and assessing organ dysfunction severity. X-rays effectively detect pulmonary infections, while ultrasound can reveal fluid collections, abscesses, and other abnormalities [27]. Computed tomography (CT) scans and magnetic resonance imaging (MRI) are useful for identifying soft tissue and bone infections [27]. However, these modalities lack specificity for sepsis and are typically used alongside blood culture analysis and biomarker testing [23,31]. Molecular diagnostic techniques like PCR and real-time PCR offer rapid pathogen identification and can potentially reduce hospitalization, ICU stays, and mortality rates by detecting pathogens missed by conventional methods [31,32,34]. While imaging is supportive, molecular diagnostics and biomarker testing provide critical insights for early sepsis diagnosis and treatment.

Microbiological cultures: Microbiological cultures, particularly blood cultures, remain the cornerstone for sepsis diagnosis, widely used to identify bloodstream pathogens [35]. Despite being mandated in US hospitals as a core sepsis measure, blood cultures have drawbacks, such as long turnaround times and reduced sensitivity in patients who have received antibiotics [35,36]. Molecular diagnostic techniques such as PCR and real-time PCR offer rapid pathogen detection capabilities and can detect a broader range of organisms than traditional methods, potentially improving patient outcomes by accelerating diagnosis and treatment initiation [30,35,37,38]. Commercial molecular multiplex technologies also promise comprehensive and fast sepsis diagnosis [36]. These advances highlight the potential of molecular diagnostics in complementing and enhancing traditional microbiological cultures for more effective sepsis management through early diagnosis and targeted treatment.

Challenges and Limitations in Diagnosis

**Figure 1:**
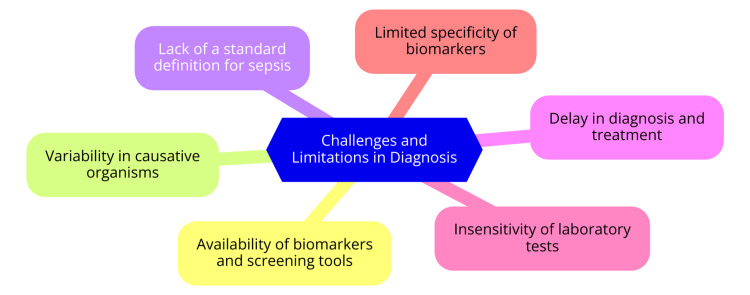
Challenges and limitations in diagnosis Image Credit: Dr. Aniket G. Pathade

Diagnosing sepsis early presents significant challenges due to its varied clinical presentation and the absence of specific symptoms in its initial stages [39]. Several factors contribute to these challenges. Firstly, biomarkers and screening tools essential for early diagnosis may not be universally accessible, particularly in resource-limited settings [40]. Secondly, the diversity of causative organisms across different infections complicates sepsis diagnosis, as each source of infection can lead to varied clinical outcomes [40]. Moreover, the absence of a standardized definition for sepsis contributes further to diagnostic complexity, impacting consistency in clinical practice and research [40]. Furthermore, delays in diagnosing and initiating treatment for sepsis are common, often occurring because clinicians may only order diagnostic tests once symptoms manifest in patients, potentially leading to critical delays [39]. Laboratory tests, such as blood cultures, also pose challenges due to their reduced sensitivity, especially in patients who have received prior antibiotic therapy [32]. Additionally, biomarkers like CRP and PCT exhibit limited specificity, which can result in both false-positive and false-negative results, further complicating accurate diagnosis [33]. Despite these obstacles, advancements in molecular diagnostic techniques and biomarker research offer promising avenues for addressing some of these limitations and enhancing early sepsis detection [32,33]. These technologies can improve diagnostic accuracy, shorten turnaround times, and provide targeted treatment strategies, potentially reducing the morbidity and mortality associated with sepsis. Challenges and limitations in diagnosis are shown in Figure 1.

Therapeutic advances in sepsis management

Antibiotic Therapy

Recent advancements in sepsis management have emphasized optimizing antibiotic therapy, focusing on targeted antibiotics and combination treatments. The research underscores the importance of initiating antimicrobial therapy within the first hour of sepsis recognition, as delays have been linked to higher mortality rates [41]. Despite these efforts, challenges such as pathogen resistance and the limited efficacy of antibiotics in reducing sepsis mortality persist [42,43]. To address these challenges, researchers are exploring novel adjunctive therapies, including synthetic antimicrobial peptides, anti-inflammatory agents, immunomodulators, and blood purification techniques, which have shown promise in providing additional therapeutic benefits [43]. Moreover, nanotechnology-based approaches such as nanoparticle-mediated drug delivery of antibiotics are being investigated. These innovations aim to combat bacterial resistance and optimize drug pharmacokinetics [43,44]. While blood culture analysis remains the established method for diagnosing sepsis, emerging molecular diagnostic technologies offer potential advancements. These technologies feature broad-based pathogen detection, high sensitivity and specificity, the ability to detect multiple pathogens, and the identification of drug-resistant strains [32]. These advancements in diagnostic and therapeutic strategies promise to enhance early sepsis detection and improve treatment outcomes.

Fluid Resuscitation

Recent insights into fluid resuscitation strategies for sepsis have prompted a reassessment of traditional approaches. While aggressive fluid administration has long been the standard practice in treating septic shock, emerging evidence has cast doubts on its effectiveness and safety. Previously, the Surviving Sepsis Campaign guidelines advocated rapid intravenous (IV) fluid administration of at least 30 mL/kg. Still, this recommendation has been downgraded due to insufficient high-quality evidence supporting its benefits [45,46]. Observational studies have shown that IV fluid resuscitation can benefit and harm patients, prompting a shift towards more personalized approaches tailored to individual patient needs [46]. There is increasing skepticism regarding large-volume fluid resuscitation, particularly in patients with severe sepsis, as it may exacerbate vasodilatory shock [47]. The concept of fluid therapy now encompasses distinct phases, resuscitation, optimization, stabilization, and evacuation, with a renewed emphasis on managing excess fluid accumulation during critical illness treatment [45]. This evolving perspective reflects a more nuanced and patient-centered approach to fluid resuscitation in sepsis, recognizing the potential risks associated with aggressive fluid management strategies.

Vasopressor Therapy

Recent research has underscored norepinephrine as the preferred first-line vasopressor for treating septic shock, owing to its favourable hemodynamic profile and lower incidence of adverse events than dopamine [48]. Additionally, vasopressin has shown promise in augmenting vasopressor therapy in septic shock by potentially reducing the need for other vasopressors, as indicated in smaller studies [49]. Vasopressors play a crucial role in septic shock by correcting vascular tone depression and enhancing organ perfusion pressure [50]. While studies have not definitively shown a survival advantage of one vasopressor over another, the choice often remains empirical, with norepinephrine recommended as the initial agent of choice [48]. Recent clinical trials have highlighted that patients with sepsis-induced hypotension who received lower volumes of IV fluids within the first 24 hours and higher doses of vasopressors achieved similar outcomes compared to those receiving higher fluid volumes and lower vasopressor doses [51]. This suggests that both vasopressor therapy and high-volume IV fluids can effectively manage sepsis, underscoring the importance of individualized treatment approaches tailored to patient-specific needs.

Immunomodulatory Approaches

Immunomodulatory strategies have emerged as potential therapies for managing sepsis. The Surviving Sepsis Campaign guidelines currently recommend corticosteroids as the sole immunomodulatory therapy for adults with septic shock who require ongoing vasopressor therapy [52]. However, efforts to target the pro-inflammatory phase of sepsis have generally not succeeded in clinical trials, necessitating new approaches that focus on modulating the host immune response in sepsis [53]. Recent research increasingly indicates that immune suppression is a critical pathophysiological factor in sepsis. Consequently, there is growing interest in immunomodulatory agents and novel therapeutics that can enhance immune cell function to mitigate this immune suppression [54]. Precision immunotherapy, which involves monitoring the immune response in sepsis and administering immunoadjuvant therapies accordingly, has shown the potential to restore immune function during sepsis [55]. Nonetheless, significant uncertainties remain regarding identifying patients who would benefit most from such treatments, the feasibility of tailoring treatments to individual immune profiles, and the optimal timing for initiating interventions during the disease [53]. These insights underscore the potential of immunomodulatory approaches in sepsis management, offering promising avenues for further research and development. Advancements in understanding immune dynamics and therapeutic interventions promise to improve outcomes for septic patients in the future.

Supportive Therapies

Patients with sepsis often require a range of supportive therapies to manage the complexities of their condition. Mechanical ventilation is frequently necessary for patients experiencing severe respiratory distress, a common complication of sepsis. This intervention provides supplemental oxygen and assists with breathing when the lungs are compromised by infection-induced inflammation. Mechanical ventilation helps maintain adequate oxygen levels in the bloodstream, supporting vital organ function and aiding the patient's recovery [38]. Renal replacement therapy (RRT) plays a crucial role in managing sepsis-related kidney failure. This therapy involves the removal of excess fluid and toxins from the body, addressing fluid overload and electrolyte imbalances. In sepsis, where renal function can be severely compromised due to systemic inflammation and decreased blood flow to the kidneys, RRT helps stabilize blood pressure and regulate body temperature. By supporting kidney function through filtration and fluid management, RRT contributes significantly to the overall management of sepsis and improves patient outcomes [56]. These supportive therapies, alongside antibiotic therapy and fluid resuscitation, form the foundational components of sepsis management protocols. However, the effectiveness of these interventions hinges on their tailored application to individual patients. Sepsis presents diverse clinical manifestations and varying organ involvement, necessitating personalized treatment strategies. Monitoring patient responses and adjusting therapies are critical to optimizing outcomes and minimizing complications associated with sepsis treatment [38].

Emerging technologies and innovations

AI in Sepsis Management

Integrating AI into sepsis management represents a transformative advancement in healthcare. AI algorithms, utilizing machine learning and deep learning techniques, have shown considerable potential in improving early detection, risk assessment, and clinical decision-making for sepsis. These algorithms can analyze extensive datasets, including electronic health records, laboratory findings, and imaging results, to identify subtle patterns that may indicate the onset of sepsis. Importantly, AI systems can continually learn and adapt, enhancing their ability to predict sepsis onset more accurately over time [9]. Beyond diagnosis, AI applications in sepsis offer predictive analytics that enables healthcare providers to anticipate patient deterioration, facilitating proactive interventions. Decision support systems powered by AI assist clinicians in devising personalized treatment plans by considering individual patient characteristics and response histories. Despite these significant advancements, several challenges remain, including concerns about data privacy, the interpretability of AI-driven insights, and the seamless integration of AI tools into clinical workflows. Addressing these challenges will be crucial to fully harnessing the potential benefits of AI in enhancing sepsis management [57].

Precision Medicine Approaches

Precision medicine, tailored to individual patient characteristics, is emerging as a promising frontier in sepsis management. This approach acknowledges the diverse and complex nature of sepsis presentations by aiming to identify specific patient subgroups defined by distinct molecular, genetic, or immunological profiles. Precision medicine can potentially optimize therapeutic interventions, reducing adverse effects and enhancing treatment outcomes [58]. Advances in genomic studies are unraveling the genetic factors that influence susceptibility to sepsis and response to treatment. Biomarker profiling provides deeper insights into the host's immune and inflammatory responses, paving the way for targeted therapies tailored to individual patient needs. Effective implementation of precision medicine in sepsis hinges on robust translational research efforts, interdisciplinary collaboration across healthcare disciplines, and the development of practical clinical tools that facilitate the application of personalized treatment strategies [59]. This holistic approach promises to improve patient care and represents a paradigm shift towards more effective and tailored management of sepsis.

Telemedicine in Sepsis Care

Telemedicine has emerged as a revolutionary force in healthcare, presenting new avenues for delivering sepsis care. Its application in sepsis management spans from remote monitoring to virtual consultations, offering opportunities for timely interventions and alleviating burdens on healthcare systems. Remote monitoring tools enable continuous tracking of vital signs and biomarkers, providing real-time data crucial for the early detection of sepsis [60]. One of the key benefits of telemedicine is its ability to facilitate rapid communication among healthcare providers, promoting collaboration and ensuring prompt decision-making in sepsis cases. Virtual consultations enhance care delivery by enabling expert input, particularly valuable in resource-limited settings where access to specialized care may be limited [60]. Despite these advantages, the widespread adoption of telemedicine in sepsis care faces challenges. Technological disparities among healthcare facilities, varying regulatory frameworks across regions, and the necessity for standardized telemedicine protocols are critical issues that must be addressed. Overcoming these challenges is essential to fully harness the potential benefits of telemedicine in improving sepsis outcomes and enhancing patient care [61].

Challenges and future directions

Antibiotic Resistance

The increasing worldwide prevalence of antibiotic resistance presents a significant obstacle in effectively treating sepsis. The excessive reliance on broad-spectrum antibiotics and their inappropriate utilization contribute to the rise of multidrug-resistant pathogens, reducing the availability of effective antimicrobial treatments. Managing antibiotic resistance in sepsis demands a comprehensive strategy that includes implementing antimicrobial stewardship programs, advancing the development of new antibiotics, and fostering global collaborations to monitor and address resistance patterns. Strategies to optimize antibiotic usage, deploy rapid diagnostic tools, and explore alternative therapeutic approaches are essential to navigating the complex landscape of antibiotic resistance. These efforts are critical for improving treatment outcomes and preserving the effectiveness of these life-saving medications [62].

Personalized Medicine Challenges

While precision medicine shows immense potential in customizing sepsis treatment based on individual patient characteristics, several challenges must be overcome. Key obstacles include identifying pertinent biomarkers, seamlessly integrating genomic data into clinical practices, and establishing standardized protocols for applying for precision medicine in sepsis care. These hurdles are critical as they affect the effective implementation and scalability of personalized approaches. Moreover, ensuring equitable access to personalized treatments and addressing ethical concerns such as data privacy and consent are crucial for the widespread adoption of precision medicine in sepsis management. These considerations underscore the importance of developing frameworks prioritizing patient welfare while leveraging advanced medical technologies. Successfully tackling these challenges will necessitate collaborative efforts across research, healthcare, and regulatory sectors. By doing so, we can unlock the full potential of precision medicine to enhance sepsis outcomes and pave the way for more effective and tailored patient care [63].

Integrating Technology Into Clinical Practice

Incorporating emerging technologies like AI and telemedicine into everyday clinical operations encounters significant implementation hurdles. Resistance to change among healthcare providers, disruptions to established workflows, and the necessity for substantial investments in technology infrastructure are prominent barriers that must be addressed. Achieving interoperability standards across various technologies, ensuring robust data security measures, and offering comprehensive training programs for healthcare personnel are essential to overcoming these challenges. These measures are crucial for fostering confidence in the reliability and usability of these technologies in clinical settings. Finding a harmonious balance between technological innovation and pragmatic implementation is key to fully harnessing the potential benefits of these tools in sepsis management and advancing overall healthcare delivery [63].

Global Health Disparities in Sepsis Management

Sepsis poses a disproportionate burden on populations in low- and middle-income countries, exacerbating existing global health disparities. Limited access to these regions' healthcare resources, diagnostic tools, and essential medications impedes effective sepsis management. Closing these gaps necessitates coordinated efforts to strengthen healthcare infrastructure, enhance education and awareness, and facilitate knowledge transfer and resources. International collaborations are crucial in addressing these disparities by advocating for equitable healthcare policies and developing cost-effective interventions. Moreover, fostering partnerships that promote sustainable healthcare solutions and support local capacity building is essential. Ensuring universal access to advancements in sepsis management requires a collective commitment to improving healthcare access and outcomes globally. By prioritizing equity and inclusivity in healthcare initiatives, we can mitigate the impact of sepsis and enhance health outcomes for all, irrespective of geographic location or economic status [63].

## Conclusions

This comprehensive review has delved into the intricate landscape of sepsis management, offering insights into predictors, diagnostic tools, and therapeutic advances. Key findings underscore the dynamic nature of sepsis as a syndrome, emphasizing the critical importance of early detection and intervention. From traditional clinical approaches to cutting-edge technologies, the multifaceted strategies explored in this review advocate for a holistic and personalized approach to sepsis care. The implications for clinical practice are profound, urging clinicians to adapt to evolving definitions, integrate advanced diagnostic tools, and embrace precision medicine. Furthermore, incorporating AI and telemedicine necessitates a commitment to training and optimization. Judicious antibiotic use and recognition of global health disparities underscore the need for a collaborative, global effort in sepsis care. Looking forward, recommendations for future research highlight the importance of longitudinal studies on biomarkers, precision medicine trials, implementation science in technology, global health interventions, and innovative antibiotic stewardship strategies. By addressing these priorities, the scientific and healthcare communities can pave the way for transformative advancements in sepsis management, ultimately enhancing patient outcomes and reducing the worldwide impact of this critical syndrome.
